# Tobacco Smoke Condensate Induces Morphologic Changes in Human Papillomavirus-Positive Cervical Epithelial Cells Consistent with Epithelial to Mesenchymal Transition (EMT) with Activation of Receptor Tyrosine Kinases and Regulation of *TGFB*

**DOI:** 10.3390/ijms25094902

**Published:** 2024-04-30

**Authors:** Zaniya A. Mark, Linda Yu, Lysandra Castro, Xiaohua Gao, Noelle R. Rodriguez, Deloris Sutton, Erica Scappini, Charles J. Tucker, Rob Wine, Yitang Yan, Evangeline Motley, Darlene Dixon

**Affiliations:** 1 Department of Biochemistry, Cancer Biology, Neuroscience, and Pharmacology, Meharry Medical College, Nashville, TN 37208, USA; 2 Molecular Pathogenesis Group, Mechanistic Toxicology Branch, National Institute of Environmental Health Sciences, NIH, Research Trriangle Park, Durham, NC 27709, USAcastro@niehs.nih.gov (L.C.); noelle.rodriguez@nih.gov (N.R.R.);; 3 Comparative and Molecular Pathogenesis Branch, Division of Translational Toxicology, National Institute of Environmental Health Sciences, NIH, Research Triangle Park, Durham, NC 27709, USA; 4 Signal Transduction Laboratory, Division of Intramural Research, National Institute of Environmental Health Sciences, NIH, Research Triangle Park, Durham, NC 27709, USA; scappinie@niehs.nih.gov (E.S.); tucker1@niehs.nih.gov (C.J.T.); wine@niehs.nih.gov (R.W.); 5 Department of Microbiology, Immunology, Physiology, Meharry Medical College, Nashville, TN 37208, USA; emotley@mmc.edu

**Keywords:** high-risk human papillomavirus (HR-HPV), cervical cancer (CC), cigarette smoke condensate (CSC), epithelial to mesenchymal transition (EMT), receptor tyrosine kinase (RTK), transforming growth factor-beta (TGFB)

## Abstract

High-risk human papillomavirus (HR-HPV; HPV-16) and cigarette smoking are associated with cervical cancer (CC); however, the underlying mechanism(s) remain unclear. Additionally, the carcinogenic components of tobacco have been found in the cervical mucus of women smokers. Here, we determined the effects of cigarette smoke condensate (CSC; 3R4F) on human ectocervical cells (HPV-16 Ect/E6E7) exposed to CSC at various concentrations (10^−6^–100 μg/mL). We found CSC (10^−3^ or 10 μg/mL)-induced proliferation, enhanced migration, and histologic and electron microscopic changes consistent with EMT in ectocervical cells with a significant reduction in E-cadherin and an increase in the vimentin expression compared to controls at 72 h. There was increased phosphorylation of receptor tyrosine kinases (RTKs), including Eph receptors, FGFR, PDGFRA/B, and DDR2, with downstream Ras/MAPK/ERK1/2 activation and upregulation of common EMT-related genes, *TGFB SNAI2*, *PDGFRB*, and *SMAD2.* Our study demonstrated that CSC induces EMT in ectocervical cells with the upregulation of EMT-related genes, expression of protein biomarkers, and activation of RTKs that regulate *TGFB* expression, and other EMT-related genes. Understanding the molecular pathways and environmental factors that initiate EMT in ectocervical cells will help delineate molecular targets for intervention and define the role of EMT in the initiation and progression of cervical intraepithelial neoplasia and CC.

## 1. Introduction

Although there have been significant changes in cancer prevention and screening in the United States in recent years, cervical cancer (CC) is known to be one of the most common causes of cancer deaths for American women [1,2,3]. A major cause of CC is the long-lasting (persistent) infection of certain types of the human papillomavirus (HPV), particularly HPV-16 or HPV-18. HPV-16 and HPV-18, the two most common high-risk HPVs (HR-HPV), are responsible for ~70% of all HPV-related CCs [4]. In contrast to low-risk HPVs, HR-HPVs express two potent oncoproteins, E6 and E7, that mediate, respectively, the degradation of cellular p53 and pRb, which are two tumor suppressor proteins essential for cell cycle control [4,5] and genome stability [4,6], thereby leading to HPV-induced carcinogenesis. In most women, the immune system clears the infection without any problems, while others may develop precancerous lesions known as cervical dysplasia or cervical intraepithelial neoplasia (CIN) [7,8,9]. Once HR-HPV infects cervical cells, it interferes with the ways in which the cells replicate, divide, and communicate with one another, causing infected cells to multiply in an uncontrolled manner which can eventually develop into CIN in combination with other not-so-well-defined cofactors [4,7,8,9]. 

When cervical cells are infected with HR-HPV, several factors increase the chance that the HPV infection will be long-lasting and lead to precancerous cervical cells, which include having a very aggressive HPV-type (HPV-16) and environmental cofactors such as tobacco smoking. Tobacco smoke is a well-established HPV cofactor for the development of cervical precancerous lesions and cancer due to the carcinogens in tobacco that can induce DNA damage in cervical cells; in addition, the effects of smoking weaken the immune system, making it less effective at fighting HPV infections [4,5]. The American Cancer Society says that women who smoke are about twice as likely as those who do not to develop CC [8,9,10]. Interestingly, studies have found measurable amounts of carcinogenic components of cigarette smoke in the cervical mucus of women smokers such as nitrosamines (NNKs), and further research revealed a positive association between the grade of CIN and the number of cigarettes smoked [11,12,13]. However, the underlying molecular mechanism(s) between cigarette smoking and CC remains unclear. Emerging evidence suggests that cigarette smoke condensate (CSC) can induce changes in the morphology and gene expression indicative of epithelial-to-mesenchymal transition (EMT) in immortalized human epithelial cells [14,15]. Smoking has been shown to activate EMT in other cancers [14,15]. This study explored the potential effects of CSC on early changes in the pathogenesis of CC using human epithelial HPV-16/E6E7 ectocervical cells to determine if exposure to CSC could induce EMT and identify key molecular markers, as well as histomorphological and functional changes that are important in CSC-induced EMT and cervical dysplasia or neoplasia.

Several distinct molecular processes are engaged in order to initiate an EMT process and enable it to reach completion. As a cell undergoes EMT, cytoskeletal changes and cell signaling pathways are altered [16,17]. Additionally, it is increasingly apparent that signaling pathways cooperate in the initiation of EMT. The EMT program is activated by multiple signaling pathways including transforming growth factor-beta (TGFB) and crosstalk with downstream target Ras/mitogen-activated protein kinase (MAPK) pathway. TGFB including its three mammalian isoforms—TGFB1, TGFB2, and TGFB3—can regulate EMTs, with distinct outcomes depending on the tissue and on the state of cell differentiation [18]. The effects of TGFB are triggered by the activation of heteromeric complexes of TGFB transmembrane type II (TBRII) and type I (TBRI) receptors which can lead to the phosphorylation of downstream Ras/MAPK/Erk1/2 target pathway [18]. The main feature of the EMT induced by TGFB is the regulation and transcription of genes, such as snail family transcriptional repressor (*SNAI*)*1*/*2* and switching from cytokeratin to mainly vimentin intermediate filaments, and an enhancement of cell migration. TGFB-activated gene suppressor of mothers against decapentaplegic (*SMAD*)*2* revealed that platelet-derived growth factor receptor (PDGFR)A/B signaling is a potential upstream regulator indicating a functional interaction between PDGFR-signaling and *SMAD2* in a TGFB-dependent fashion [19]. The increased expression of the PDGF family by TGFB has been well documented in various cell types, generally indicating a more aggressive biological behavior than those with low or normal expression [19,20]. The markers of TGFB-induced EMT also include the delocalization of E-cadherin from cell junctions [18,21]. TGFB signaling interacts with receptor tyrosine phosphorylation (RTKs) pathways and their interplay is important for cancer progression and development [21]. TGFB and RTK activation appears to be the critical checkpoint controlling both *SMAD* and non-*SMAD* signals, which crosstalk with various transcription and signal transduction pathways regulating their activities or expression. The EMT program is also activated by several epigenetic and post-translational modifications such as RTKs. RTKs are generally activated by growth factor receptor-specific ligands. Growth factor ligands bind to the extracellular regions of RTKs, and the receptor is activated by ligand-induced receptor dimerization and/or oligomerization [16,22]. However, the effects of short-term exposure to CSC on cervical epithelial cells need to be further explored to elucidate the potential mechanism(s) of the RTK/MAPK/TGFB signaling pathway in CC development and progression. This study sheds light on the role of environmental factors that promote CC development and the molecular mechanism(s) of EMT-associated pathways, which is important in understanding CC initiation and progression.

Our aim was to determine the potential effects of CSC on human ectocervical (HPV16 Ect1/E6E7) cells on EMT induction, cell morphology, motility, gene expression, and RTK signaling. We hypothesized that HPV-immortalized cervical cells exposed to CSC can transition from an epithelial to mesenchymal phenotype causing functional and morphological changes that express EMT markers and EMT-related genes with activation of RTKs important in EMT signaling. Our findings will further help define the molecular mechanism(s) by which tobacco smoke condensate exposure in human ectocervical cells induces EMT and better understand the role of EMT in the initiation and progression of CIN and CC.

## 2. Results

### 2.1. CSC Induced Proliferation of Human Cervical Epithelial Cells

**Figure 1:**
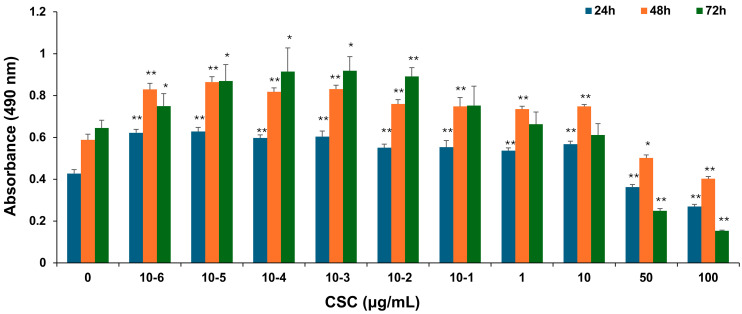
CSC-induced proliferation in human cervical epithelial cells. HPV-16 Ect1/E6E7 cells were treated with CSC at 10^−6^-100 μg/mL for 24 h, 48 h, and 72 h, respectively. The proliferative effects were measured by an MTS assay. Data are represented as mean ± SE; * *p* < 0.05 and ** *p* < 0.01 compared to the control group.

To explore the potential effects of CSC on human cervical epithelial cells, HPV-16 Ect1/E6E7 cells were exposed to various concentrations of CSC for 24 h, 48 h, and 72 h, and cell proliferation was examined by an MTS assay as described above. HPV-16 Ect1/E6E7 cells were significantly increased by CSC administration at concentrations of 10^−6^–10 μg/mL. CSC significantly decreased proliferation at concentrations of 50 and 100 μg/mL at 24 h, 48 h, and 72 h, indicating an inhibitory effect at those concentrations on the cells. At 24 h and 48 h, we saw a significant stimulatory effect on the HPV-16 Ect/E6E7 cells at concentrations of 10^−6^–10 μg/mL compared to 72 h, whereby a significant increased cellular proliferation was only observed at 10^−6^–10^−1^ μg/mL, although a concentration-dependent trend towards enhanced proliferation was observed at 1 μg/mL, but not at 10 μg/mL (Figure 1). Overall, these results support that CSC can promote the proliferation of human cervical epithelial cells.

### 2.2. CSC-Induced Early EMT Morphologic Changes in Human Cervical Epithelial Cells

**Figure 2:**
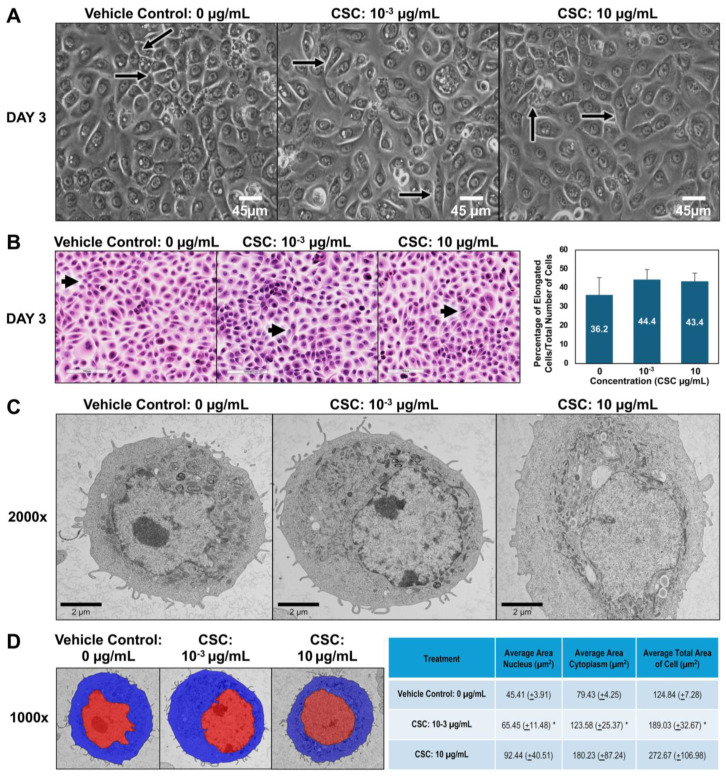
CSC-induced early EMT in human cervical epithelial cells. HPV-16 Ect1/E6E7 cells were treated with CSC at concentrations 10^−3^ μg/mL and 10 μg/mL for 72 h to observe EMT morphologic changes. Cell morphology was examined by the following: (**A**) brightfield microscopy; note elongated cells in treated groups (arrows). (**B**) Light microscopy using hematoxylin and eosin (H&E) staining; note elongated cells in treated groups (arrows). (**C**) Transmission electron microscopy (TEM). (**D**) Overlay TEM images. Brightfield images = ×40 magnification; light microscopy images = ×20 magnification. TEM images ×2000. Visiopharm overlay images; the blue label is the cytoplasm, and the red label is nucleus= ×1000. * Data are represented as mean ± SE; * *p* < 0.05 compared with the control group.

To further explore the potential effects of CSC on human cervical epithelial cells. We observed the morphological and functional changes equivalent to early EMT characteristics at 24, 48, and 72 h. Using brightfield microscopy, we wanted to see if there was a difference in the size and shape of cells compared to controls. In our HPV-16 Ect1/E6E7 cells, we found that the cells exposed to CSC at concentrations of 10^−3^ and 10 μg/mL became more elongated at 72 h, gaining a mesenchymal or spindle-like morphology compared to controls which had more of a “cobblestone” appearance (Figure 2A). We further confirmed these findings by light microscopy. We observed at 72 h that HPV-16 Ect1/E6E7 cells undergoing EMT had phenotypic changes ranging from a rounded appearance to a spindle-shaped cell visible as a longitudinally elongated cell type with a mesenchymal appearance when CSC was added compared to the controls (Figure 2B). To further highlight that the cells were undergoing early EMT, we were able to show a trend in the increase of elongated cells when CSC was added compared to controls, although it was not significant. Next, we wanted to observe the ultrastructural characteristics of cells, so we examined the subcellular organelles of the cells. At the ultrastructural level, the HPV-16 Ect1/E6E7 cells showed a high nuclear–cytoplasm ratio with abundant electron-lucent cytoplasm with relatively few cellular organelles but with prominent polyribosomes when treated with CSC. CSC-treated cells were enlarged compared to the controls and had decreased surface filopodia and cytoplasmic swelling, and variable-sized mitochondria were observed in HPV-16 Ect1/E6E7 at 72 h (Figure 2C). Ultrastructural changes consisting of nuclear/cytoplasm ratio showed CSC-treated cells were enlarged nearly 1.5 times than that of controls at doses of 10^−3^ and 10 µg/mL, but it was significant at a dose of 10^−3^ µg/mL (Figure 2D).

### 2.3. CSC Induced Expression of Biomarkers of EMT in Human Cervical Epithelial Cells

**Figure 3:**
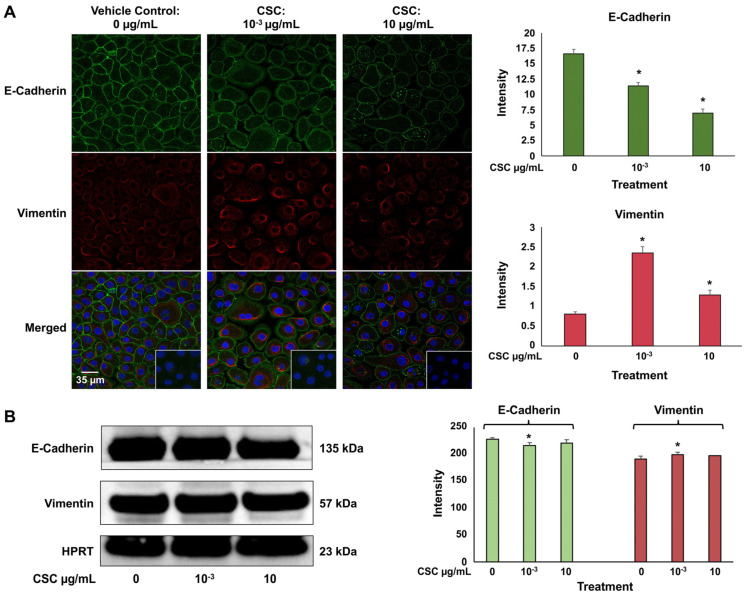
CSC-induced expression of early EMT genes and protein biomarkers in human cervical epithelial cells. HPV-16 Ect1/E6E7 cells treated with CSC at 10^−3^ μg/mL and 10 μg/mL for 72 h. (**A**) Immunofluorescence and (**B**) Western blotting analysis were used to analyze protein expressions of EMT-related markers in human cervical epithelial cells exposed to CSC. Data are represented as mean ± SE; * *p* < 0.05 compared with the control group.

We used immunofluorescence staining to identify an epithelial cell marker E-cadherin, which is an adhesion molecule that is expressed on the plasma membrane, and mesenchymal marker vimentin, which is an intermediate filament expressed in the cytoplasm. In the HPV-16 Ect1/E6E7 cells at 72 h, we saw a significant decrease in E-cadherin expression at both concentrations of 10^−3^ μg/mL and 10 μg/mL; however, vimentin expression at both concentrations of CSC was significantly increased in the HPV-16 Ect1/E6E7 cells (Figure 3A). At 72 h, we also saw a trend indicating a decrease in E-cadherin expression with an increase in vimentin expression at both concentrations of 10^−3^ μg/mL and 10 μg/mL in the HPV-16 Ect1/E6E7 cells by Western blot analysis (Figure 3B). Immunofluorescence staining and Western blotting analysis were conducted to confirm that the cells were undergoing EMT when exposed to CSC at 10^−3^ μg/mL and 10 μg/mL for 72 h in human cervical epithelial cells since this was the time point at which we saw histologic and ultrastructural cellular evidence of EMT.

### 2.4. CSC Induced Increased Migration of Scratch Assay in Human Cervical Epithelial Cells

**Figure 4:**
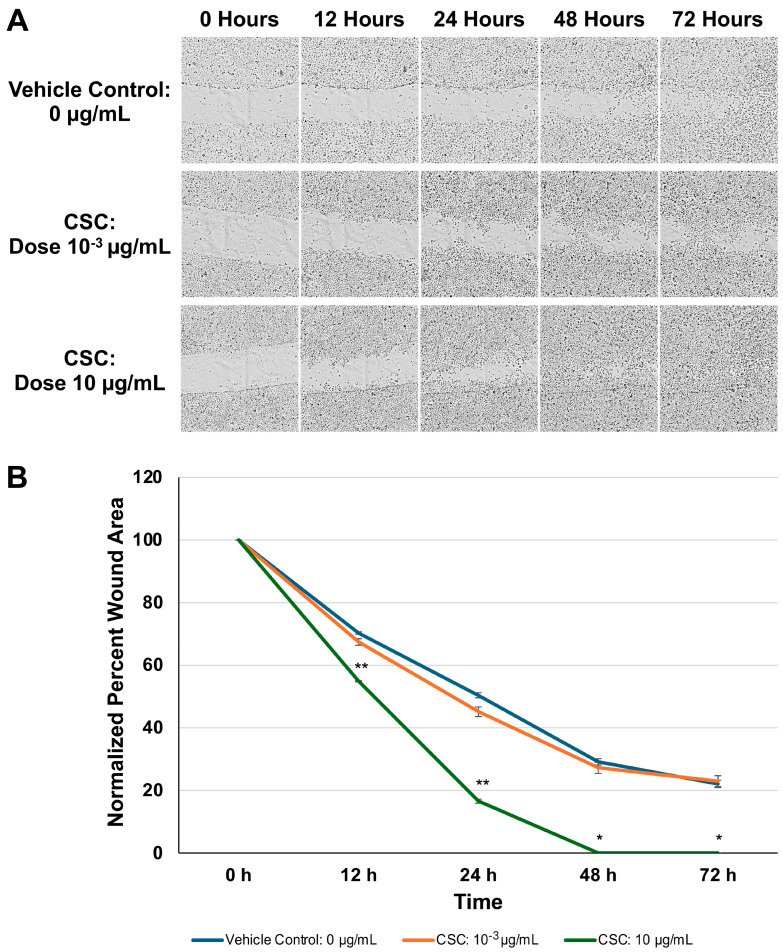
Functional analysis of CSC on HPV-16 Ect1/E6E7 cells was analyzed by (**A**) a scratch assay for 72 h. (**B**) Data are represented as percent wound area to compare the difference between the area and the average length obtained with a with the aid of a plugin under Labkit in ImageJ/Fiji (refer to methods). Data are represented as mean ± SE * *p* < 0.05, ** *p* < 0.01 compared with the control group.

To further assess if changes observed in the morphology and expression of EMT biomarkers would impact cell functions and motility, we evaluated the HPV16 Ect1/E6E7 cells following exposure to CSC using a wound healing assay (Figure 4A). In our HPV-16 Ect1/E6E7 cells, we found that the CSC-exposed cells at 10 μg/mL showed enhanced migration at 72 h compared to the controls (Figure 4B). These data suggest that CSC induces enhanced motility in human cervical epithelial cells undergoing morphological and functional changes in EMT.

### 2.5. CSC Effects on Expression of EMT-Related Genes and RTK Activation Upregulating Downstream Effector Protein, MAPK, in Human Cervical Epithelial Cells

**Figure 5:**
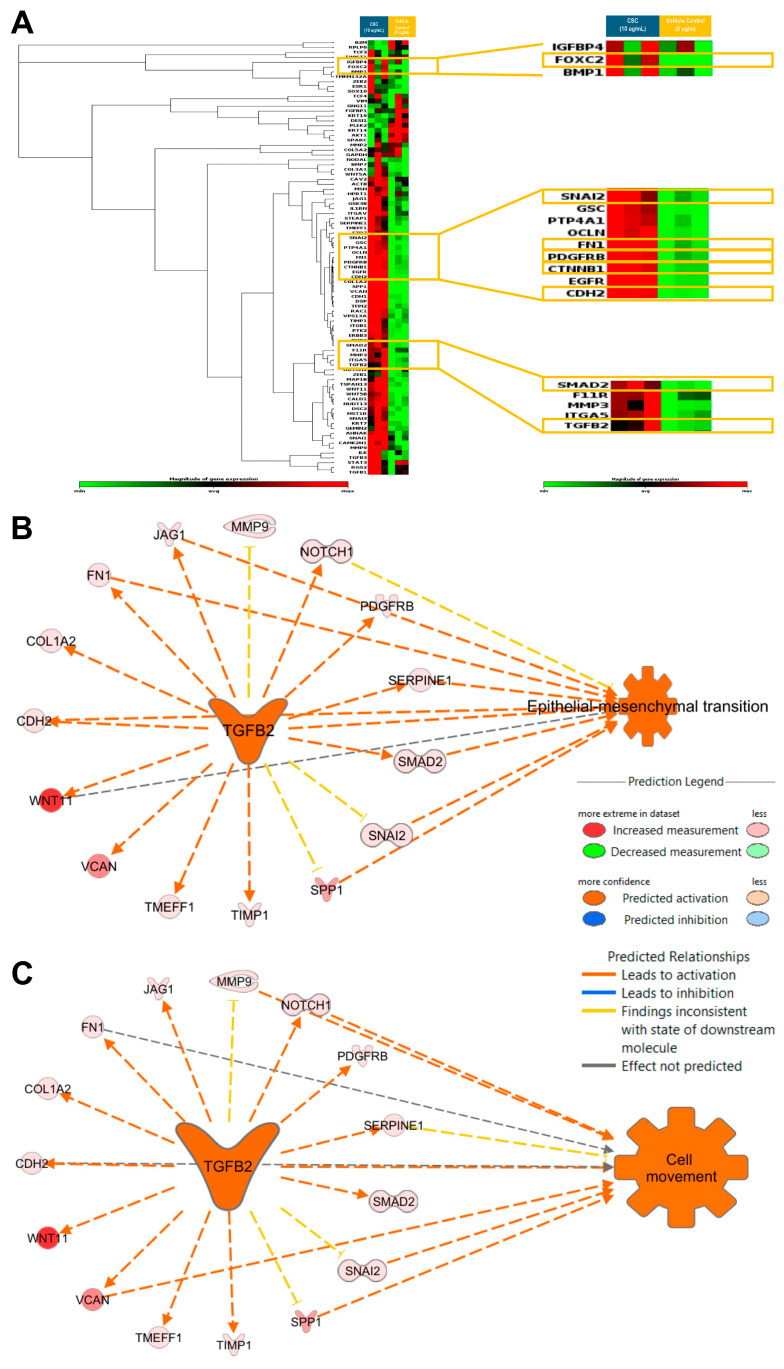
CSC effects on the expression of EMT-related genes in human cervical epithelial cells. HPV-16 Ect/E6E7 cells were exposed to 10 μg/mL CSC for 72 h to observe the expression of EMT-related genes. Upstream functional analysis of CSC-activated *TGFB2* network in an EMT RT^2^ profiler array (**A**) heat map. Ingenuity Pathway Analysis (IPA) predicted *TGFB2* to be a master upstream regulator of (**B**) EMT and (**C**) cell movement with upregulation of common EMT-related genes. Yellow boxes highlight the common EMT-genes differentially expressed in human cervical epithelial cells upon CSC treatment.

Gene expression profiling to identify EMT characteristics at a transcriptional level was carried out in the HPV-16 Ect1/E6E7 cells exposed to CSC. We assessed the expression of known EMT-related genes in an EMT RT^2^ profiler array. We observed that CSC at 10 μg/mL at 72 h significantly upregulated the EMT-related genes *TGFB2*, *SNAI2*, *SMAD2*, and *PDGFRB*, all of which were predicted to be involved in cell movement and EMT (Figure 5A; Supplemental Table S1). We found that the significantly upregulated EMT-associated genes in CSC-treated cells *TGFB2* was predicted by IPA to be a master upstream regulator of EMT and cell movement with the upregulation of common EMT-related genes such as *SNAI2*, *SMAD2*, *CDH2*, *FN1*, and *PDGFRB* (Figure 5B,C).

**Figure 6:**
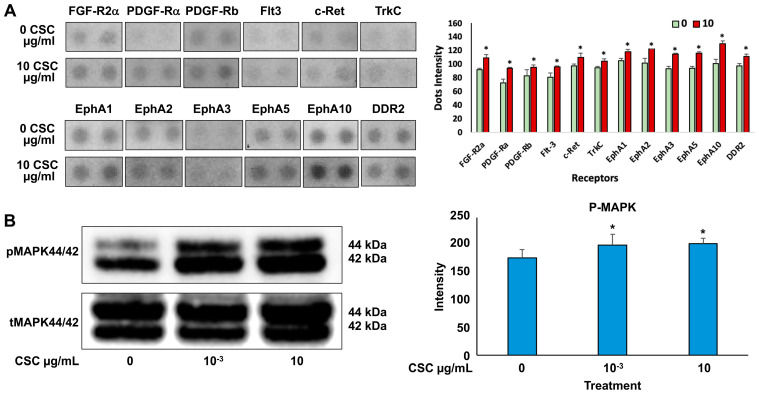
CSC effects on the expression of RTK activation upregulating downstream effector protein, MAPK, in human cervical epithelial cells. (**A**) Expression of growth factor RTKs in HPV-16 Ect/E6E7 cells shown by representative RTK array (top) and as quantitated dot blot intensity (0 and 10 µg/mL) values (mean + SE) at the bottom. (**B**) Western blotting for phosphorylated MAPK44/42. Data are represented as mean ± SE; * *p* < 0.05 compared with the control group. Significance was expressed as mean ± SE of at least three replicates.

Cells exposed to 10 μg/mL CSC showed the most significant functional and morphological characteristics of early EMT; therefore, we wanted to determine the effects of CSC on RTK activation and downstream signaling through pathways like Ras/MAPK/ERK1/2 in human cervical epithelial cells at 10 μg/mL using an RTK array. We found that HPV-16 Ect1/E6E7 CSC-exposed cells undergoing early EMT changes at 72 h showed activation of RTKs. Specifically, we observed increased activation of EPH receptors (EphA 1, 2, 3, 5, and 10), FGFR, PDGFRA, FLT3, c-RET, TrkC, and DDR2 in CSC-exposed cells compared to controls (Figure 6A), all of which induced the upregulation of *TGFB* and other common EMT genes such as *SNAI1* and *SNAI2*, *CTNNB1*, and *FOXC2,* which were significantly overexpressed in the CSC-treated ectocervical cells compared to controls at 72 h. To further analyze the downstream signaling pathway involvement in HPV-16 Ect1/E6E7 CSC-exposed cells at both concentrations of 10^−3^ and 10 μg/mL, we evaluated the expression of the Ras/MAPK/ERK1/2, which is known to be a key regulator of upstream RTK activation and regulator of TGFB signaling. We found increased phosphorylation of downstream effector protein MAPK/ERK1/2 in CSC-exposed cells specifically at a concentration of 10 μg/mL compared to controls by Western blotting analysis (Figure 6B).

## 3. Discussion

Cervical cancer (CC) is a highly heterogeneous disease, consisting of a distinct variety of genetic, epigenetic, and morphologically diverse cells [23,24,25,26]. A growing list of molecular and environmental cues can initiate EMT [27,28]. Tobacco smoke contributes to a greater cancer incidence and worse prognosis of CC [13,15]. Nearly all CC cases develop from cells in the ectocervix with pre-cancerous changes known as cervical intraepithelial neoplasia (CIN), which can be exacerbated by tobacco use [14]. Smoking is an established inducer of EMT, which usually operates by activating or mediating the secretion of a cohort of RTKs that induce the EMT process via the activation of their respective signaling pathways [29]. Here, we highlight findings that demonstrate cancer causation is multifactorial and provide evidence that an external risk factor, such as smoking, can result in morphological and functional changes that can lead to cell proliferation, enhanced cell motility, and activation of RTKs and TGFB-mediated signaling pathways important in EMT.

The cellular impact of carcinogens found in tobacco components varies depending on the concentration of treatment and cell type, so we chose CSC concentrations to investigate the effects of CSC on HPV-16 Ect/E6E7 cells [30]. Approximately 100% of CC patients are positive for HPV infections according to epidemiological studies, with HPV-16 being the most common HPV type involved in cervical disease and cancer [1,2]. Additionally, nearly all CC cases develop in the ectocervix (up to 90%) [2]. Therefore, we chose HPV-16 Ect/E6E7 as the most clinically relevant cell line to use to conduct our studies. We compared the most current average nicotine content in a cigarette and used it as a reference point to choose our reference cigarette for the CSC used in these studies. We chose CSC 3R4F because the constituents used to develop this reference cigarette were the most equivalent to the constituents in an average cigarette. NNK concentrations for smokers ranged from 11.9 to 115.0 ng/g in the cervical mucus [31]. The amount of NNK in the 3R4F CSC is 97.9 ng (0.0979 mg), which is within the average range concentration found in the cervical mucus [11,31]. Therefore, HPV-16 Ect/E6E7 cells were exposed to CSC at various concentrations of 10^−6^–100 μg/mL. In this study, CSC was found to induce proliferation of human epithelial cervical cells at 10^−6^–10 ug/mL, but inhibitory effects were observed at 50 and 100 mg/mL. We also evaluated the effects of CSC at a low and higher concentration (10^−3^ μg/mL and 10 μg/mL, respectively) at 72 h during the induction of early EMT characteristics in HPV-16 Ect/E6E7 cells. It has been reported that the morphological features of cervical cells are important, as the cells can behave differently to the various concentrations of CSC to which they are exposed [14,26]. In this study, we found the following morphological changes in cervical epithelial cells with respect to EMT characteristics and disease progression at 72 h that included nuclear enlargement with increased variation in size and shape. It has been reported that the amount of cytoplasm in relation to the size of the nucleus (nuclear–cytoplasmic ratio) is one of the most important bases for identifying early EMT and assessing dysplastic cells to determine the grade of CIN [7,8,9]. Increased ratios have been known to be associated with more severe degrees of CIN [9].

EMT is a biological process critical in cancer cell metastasis and cancer stem cell formation [32,33,34]. It allows a polarized epithelial cell, which normally interacts with the basement membrane via its basal surface to undergo multiple biochemical changes that enable it to assume a mesenchymal cell phenotype, which confers enhanced migratory capacity, invasiveness, elevated resistance to apoptosis, and greatly increased the production of ECM components [33]. This biological process can be reversed, whereby mesenchymal cells can become epithelial cells, and this can occur during normal development, in pluripotent stem cell reprogramming and cancer metastatic events. During EMT, there is a loss of epithelial cell markers, such as cytokeratins and E-cadherin, followed by an upregulation in the expression of mesenchymal cell markers, such as N-cadherin, vimentin, and fibronectin [33,34]. These changes in epithelial and mesenchymal cell marker expressions lead to a reduction in the adhesion between the transitioning and adjacent epithelial cells and an increase in the secretion of enzymes that degrade the extracellular matrix [33,35]. Collectively, this results in epithelial cells losing apical–basal cell polarity, reorganizing their cytoskeleton, and reprogramming their gene expression, which can allow for the development of an invasive phenotype in cancer metastasis or cancer stem cells that have unique self-renewal capabilities and differentiation and proliferation properties thought to be essential for cancer initiation [33,34,36,37,38]. Molecular links have shown EMT programming plays a critical role in cancer initiation and in both the early and late phases of cancer metastasis [18,26,38]. EMT has been classified into three categories: type I, type II, and type III, which have very different functional consequences. Type I occurs during embryogenesis where cells need to migrate to adjacent tissues in order to form new organs and tissues [33,37,39]. Type II is associated with wound healing, whereby fibroblasts repair or rebuild tissues [16,33]. Unlike types I and II which perform necessary physiologic functions, type III is a pathophysiologic adaptation of the process and is closely associated with the initiation and progression of neoplasia occurring in cells containing certain epigenetic and genetic changes [16,33,35,39,40,41,42,43,44]. In this study, we focused on uncovering the cellular and molecular mechanisms whereby cigarette smoke condensate (CSC) could induce EMT programs that occur in type III EMT.

Among the EMT inducers, TGFB receives substantial attention, largely because of its potency in inducing EMT in cell culture and its roles in cancer-associated EMT, while TGFB family proteins also direct EMT during development [18,21]. Consequently, TGFB-induced EMT has been better characterized than EMT in response to other inducers and often serves as a paradigm for the analyses of this process [38]. In TGFB signaling, members of the TGFB superfamily are the primary factors that drive EMT which include TGFB three isoforms (TGFB1, TGFB2, and TGFB3). In human malignancies, TGFB1 has been shown to drive the induction of EMT by activating the transcription of genes, such as *SNAI1/2* and *CDH2*, which are important in switching from E-cadherin to N-cadherin, and the expression of mainly vimentin intermediate filaments with enhancement of cell migration [27,38,39]. In exposing HPV-16 Ect1/E6E7 cells to CSC, we found upregulation of *TGFB*, with significant overexpression of other EMT-related genes like *SNAI2*, *SMAD2*, *CDH2*, and *PDGFRB* needed for enhanced cell motility. In this study, *TGFB2* appeared to be the isoform significantly upregulated, although isoforms 1 and 3 were increased but not significantly. The markers of TGFB-induced EMT include the delocalization of E-cadherin from cell junctions [18,21]. This further explains why we saw significant morphological and cytoskeletal changes, including the induction of a mesenchymal-like cell phenotype with decreased E-cadherin and increased vimentin expression in HPV-16 Ect/E6E7 ectocervical cells at 72 h following CSC exposure.

RTKs, such as epidermal growth factor receptor (EGFR), fibroblast growth factor receptor (FGFR), vascular endothelial growth factor receptor (VEGFR), and PDGFR, activate various signaling pathways, including those mediated by the Ras, MAPK/ERK1/2 signaling pathway [22,40]. These signaling cascades activate transcription factors (TFs) that bind to the promoters of genes that encode EMT-inducing transcription factors, such as *SNAI1/2*, *ZEB1/2*, and *TWIST1*, which induce EMT by inhibiting the expression of genes encoding cell adhesion molecules [22]. The increased expression of the RTKs, Erythropoietin-producing hepatoma (EPH) receptors, fibroblast growth factor receptor (FGFR) family, PDGFRA/B, FMS-like tyrosine kinase 3 (*FLT3*), c-Rearranged during transfection (c-RET), Tropomyosin receptor kinase C (TrkC), and discoidin domain receptor (DDR) family (DDR2) have been implicated in EMT and reported to induce morphological and cytoskeletal changes including the induction of a mesenchymal-like phenotype in cells that are E-cadherin–negative and vimentin-positive [22,31,41,42,43]. RTKs have also been found to regulate gene reprogramming, inducing an invasive behavior of cells that is reminiscent of type III EMT and associated with the upregulation of common EMT genes such as *SNAI1/2*, *PDGFRA/B*, and *SMAD2* [19,27,28]. Furthermore, the increased expression of the RTKs initiates diverse signaling pathways downstream of Ras/MAPK [29]. The role of these RTK family members in the tumorigenesis of the uterine cervix remains poorly understood.

In early EMT, TGFB interacts with RTKs and elicits signaling through both SMAD and non-SMAD signaling pathways, with crosstalk between the various signal transduction pathways and at multiple levels to provide context-dependent outcomes which include the Ras/MAPK/ERK1/2 pathway. Our results clearly demonstrate that CSC-exposed cervical epithelial cells showed morphologic evidence of EMT with the activation of five EPH receptors (EPH A1, 2, 3, 5, and 10), FGFR, PDGFRA/B, *FLT3*, c-RET, TrkC, and DDR2) which have been identified as key regulators of early EMT and regulators of common EMT genes such as *SNAI1*, *CTNNB2*, *CDH2* and *FOX* family (*FOXC2*) [27,28,30]. RTK activation leads to downstream MAPK/ERK1/2 signaling, and MAPK/ERK1/2 pathway has been shown to regulate TGFB [21]. This study demonstrates the interplay between TGFB signaling and RTKs and their influence on EMT which might possibly play a role in the progression of EMT changes from early CIN to late CIN and CC development.

In conclusion, during the precancerous lesions of the cervix, multiple factors such as growth factors, along with the activation of RTKs and signaling pathways, influence a phenotypic change in which HPV-16 Ect/E6E7 cervical epithelial cells tend to transition into a mesenchymal cell phenotype in response to CSC exposure. CSC exposure upregulated *TGFB*, increased the activation of RTKs with HPV-16 Ect/E6E7 cervical epithelial cells showing characteristics of early EMT with an increase of elongated cells when CSC was added compared to the controls, increased expression of vimentin, decreased E-cadherin expression, and enhanced cell migration and proliferation. Whether prolonged CSC exposure can induce late EMT with increased significance of elongated cells to develop a more aggressive phenotype leading to the development of CIN and CC needs further investigation. The limitations of this study include the absence of an HPV-negative control group for these experiments in addition to a lack of further investigation of the role of apoptosis in explaining the inhibitory effects observed in HPV-16 Ect1/E6E7 cervical epithelial cells exposed to higher concentrations of CSC (50–100 μg/mL). We plan to further address the long-term effects of CSC on cervical epithelial cells and EMT change and further explore the potential importance of the RTK/MAPK/TGFB signaling pathway in CIN initiation and potentially CC development and progression. Understanding the molecular and environmental factors that initiate EMT in HPV-16 Ect/E6E7 will help delineate pathways important in the progression of cervical epithelial cells to CIN and CC, and hold promise for the development of valuable biomarkers for the prognosis of CIN and CC progression and provide new ideas for more integrative therapeutic approaches for this disease.

## 4. Materials and Methods

### 4.1. Cells and Reagents

The human epithelial HPV-16 E6/E7-transformed human ectocervical epithelial cell line (HPV-16 Ect1/E6E7) was purchased from the American Type Culture Collection (ATCC# CRL-2614, Manassas, VA, USA). Cells were incubated in EpiLife Medium with 60 μM of calcium (GIBCO-BRL, MEPI500CA, Grand Island, NY, USA) supplemented with Human Keratinocyte Growth Supplements (HKGS) (GIBCO-BRL, S0015, Grand Island, NY, USA): bovine pituitary extract (BPE): 0.2% *v*/*v*; recombinant human insulin-like growth factor-I: 0.01 µg/mL; hydrocortisone: 0.18 µg/mL; bovine transferrin: 5 µg/mL; human epidermal growth factor: 0.2 ng/mL, maintained at 37 °C with 5% CO_2_ atmosphere. For subculture, the cells were passaged using trypsin for 2–3 min and maintained with a new EpiLife medium with HKGS.

### 4.2. Cigarette Smoke Condensate

The cells were treated with cigarette smoke condensate (CSC, 40 mg/mL, 3R4F cigarette, Batch R060411, nicotine level 0.16 mg/cigarette, and 1 mL/vial in DMSO), which was purchased from Murty Pharmaceuticals (Lexington, KY, USA) and diluted in culture medium to working concentrations. DMSO 0.1% was used as a vehicle control.

### 4.3. MTS Assay Kit (Cell Proliferation) (Colorimetric)

HPV-16 Ect1/E6E7 cells were plated into 96-well plates with a density of 30,000 cells per well and maintained in a culture medium. After treatment with various concentrations of CSC, 3R4F (0, 10^−6^,10^−5^, 10^−4^, 10^−3^, 10^−2^, 10^−1^, 1, 10, 50, and 100 μg/mL) for 24 h, 48 h, and 72 h, cell proliferation was evaluated using an MTS Cell Titer 96^®^ Aqueous One Solution Cell Proliferation Assay (Promega Madison, WI, USA). Absorbance was measured at 450 nm by a microplate reader SpectraMax M5 spectrophotometer (Molecular Devices, San Jose, CA, USA). The experiment was repeated in triplicate.

### 4.4. Light Microscopy: Hematoxylin & Eosin (H&E) Staining

HPV-16 Ect1/E6E7 cells were grown in chamber slides and treated with CSC; 3R4F (10^−3^ and 10 μg/mL) or DMSO 0.1% was used as a vehicle control, and cells from all respective groups were collected at 72 h and placed in 10% neutral buffered formalin for 10 min. Fixed cells were stained with H&E for 20 s, rinsed in distilled water, and dehydrated through ascending grades of alcohol to xylene and cover slipped using a permanent mounting medium. Subsequently, the stained slides were examined under a light microscope to confirm cellular architecture. Slides stained for H&E were cleaned with an isopropanol solution to prepare for digital slide scanning. The slides were then scanned using the Leica Biosystems Aperio AT2 Digital Whole Slide Scanner (Leica Biosystems, Inc., 1700 Leider Lane, Buffalo Grove, IL, USA). After scanning, the resulting digital images were viewed using Aperio^®^ Image Scope v. 12.0.1.5027 (Aperio Technologies, Inc., 1360 Park Center Dr, Vista, CA, USA), a viewing program designed to display and capture digitally scanned images. Images were segmented first using the Labkit plugin in ImageJ/Fiji (NIHV 2.9.0/1.54b, National Institutes of Health, USA), further tuned with Adjustable Watershed (setting of 2), and then the Analyze Particles feature of ImageJ/Fiji was used to extract the shape descriptor measurement of circularity for each cell where a measurement of 1.0 indicates a perfect circle; as the values approach 0.00, it indicates an increasingly elongated polygon. Cells were counted as elongated at measurements between 0.00 and 0.50 [23]. Data were exported to Microsoft Excel (https://www.microsoft.com/ko-kr/microsoft-365/excel, accessed on 22 March 2024).

### 4.5. Transmission Electron Microscopy (TEM)

HPV-16 Ect1/E6E7 cells were maintained in a culture medium until 70% confluent and then exposed to their appropriate concentrations of CSC: 3R4F (10^−3^ and 10 μg/mL) for 72 h. DMSO 0.1% was used as a vehicle control. The cells were detached with 0.25% trypsin solution (Life Technologies CAT# 25200-056, Carlsbad, CA, USA), resuspended in 1 × PBS, and centrifuged at 1000 rpm for 2 min. The supernatant was removed, and the cell pellets were fixed in 4F:1G fixative buffer [31,45]. The cell pellets were embedded in 3% water agar following rinses in phosphate buffer. Samples were post-fixed in 1% osmium tetroxide in phosphate buffer, rinsed in water, and dehydrated in an ethanolic series culminating in acetone. The samples were then infiltrated with Poly/Bed 812 epoxide resin. After polymerization, selected blocks were trimmed and semithin sections (approximately 0.5 µm thick) were cut, mounted on glass slides, and stained with 1% toluidine blue O in 1% sodium borate. Ultrathin sections (80–90 nm thick) were cut from selected blocks, placed onto 200 mesh copper grids, and then stained with uranyl acetate and lead citrate. Digital images were captured with an AMT XR 16-megapixel midmount camera (AMT, Woburn, MA, USA) attached to a Hitachi H7600 transmission electron microscope (Hitachi High-Tech Corporation, Tokyo, Japan) operating at an accelerating voltage of 80 kV, or an AMT XR 16-megapixel midmount attached to a JEOL JEM-1400+ transmission electron microscope (Tokyo, Japan) operating at an accelerating voltage of 80 kV.

### 4.6. Cytoplasmic and Nuclear Compartment Measurement Methods

TEM images of HPV-16 Ect1/E6E7 cells, captured at 1000×, were imported into the Visiopharm platform using a calibration of 101.49 pixels/µm. Calibration was verified by measurement of a scale bar. Three cells per concentration and time point were randomly selected for cytoplasmic and nuclear measurements. These cells were first identified in the imported images taken at 700× magnification, using a calibration of 84.62 pixels/µm and enclosed in a region of interest (ROI) for analysis. Three HPV-16 Ect1/E6E7 cells treated with DMSO 0.1% (vehicle control) or CSC (3R4F (10^−3^, and 10 μg/mL) at 72 h were randomly selected, one from each image provided. A total of 3 cells at a TEM magnification of 1000× were evaluated. The cytoplasm (excluding cytoplasmic projections) and nucleus were manually delineated, and the total area of the cell was calculated within Visiopharm. Data were exported to Microsoft Excel.

### 4.7. Protein Extractions and Western Blot Analysis

HPV-16 Ect1/E6E7 cells were grown in basal medium with Human Keratinocyte Growth Supplement (HKGS) treated with CSC (3R4F (10^−3^ and 10 μg/mL)and collected at 72 h in lysis buffer with proteinase inhibitors (10 μg/mL aprotinin, 10 μg/mL leupeptin, and 2 μg/mL phenylmethylsulphonyl fluoride). DMSO 0.1% was used as a vehicle control. The samples were then centrifuged at 12,000 r/min for 20 min at 4 °C. The protein lysates were collected and stored at −80 °C for Western blotting analysis or RTK arrays (see below). Protein concentrations were determined by Pierce^TM^ Bicinchoninic Acid (BCA) Protein Assay Kit (Thermo Scientific CAT# 23225, Waltham, MA, USA).

Western blotting was conducted to determine the protein expression of EMT markers and MAPK protein in HPV-16 Ect1/E6E7 cells exposed to concentrations of CSC (3R4F (10^−3^ and 10 μg/mL)) for 72 h. DMSO 0.1% was used as a vehicle control. The primary antibodies were E-Cadherin Rabbit monoclonal antibody (1:1000, Cell Signaling, #31950, Danvers, MA, USA), Vimentin Rabbit monoclonal antibody (1:1000, Cell Signaling, #5741S Danvers, MA, USA), MAPK Rabbit monoclonal antibody (1:1000, Cell Signaling, #9101), and HRP-labeled anti-human HPRT antibody (1:1000, Santa Cruz, Dallas, TX, USA). To determine the protein expression of E-Cadherin (Cell Signaling, #3195S, Danvers, MA, USA), Vimentin (Cell Signaling, #5741S, Danvers, MA, USA) and MAPK (Cell Signaling, #9101), equal amounts of protein (30 μg) obtained from vehicle- and CSC-treated cells were loaded on a 10% polyacrylamide gel (SDS-PAGE) and electrophoresed, followed by transfer to a nitrocellulose membrane. The membrane was blocked in 5% bovine serum albumin and incubated with primary antibodies above (1:1000 dilution) overnight at 4 °C. The next day, the membrane was washed and incubated with anti-mouse or anti-rabbit secondary antibodies (1:2000 dilution). Membranes were washed three times in TBS-T and incubated with secondary anti-IgG-labeled peroxidase (BD Pharmingen, San Diego, CA, USA). After washing three times in TBS-T, immune complexes were detected using the Super Signal West Pico PLUS Chemiluminescent Substrate (Protein Biology, Thermo Scientific; CAT# 34580, Waltham, MA, USA) according to the manufacturer’s instructions. HPRT was used as an internal loading control to normalize the expression of all proteins. Band intensity was quantified by ImageJ/Fiji (NIHV 2.9.0/1.54b, National Institutes of Health, USA) [24]. 

### 4.8. Confocal Immunofluorescence Staining

Immunofluorescence (IF) staining was used to detect EMT markers expression and localization in CSC-exposed HPV-16 Ect1/E6E7 cells. HPV-16 Ect1/E6E7 cells treated with CSC (3R4F (1 × 10^−3^, and 10 μg/mL)) for 72 h were fixed and incubated with primary antibodies to E-Cadherin rabbit monoclonal antibody (1:200, Cell Signaling, #31950) and Vimentin mouse monoclonal antibody (1:500, Santa Cruz, sc-6260) at 4 °C overnight, followed by incubation with Alexa Fluor 488 goat anti-Mouse (red fluorescence) secondary antibody (1:5000, Molecular Probes^®^, Eugene, OR, USA, A11001) and Alexa Fluor 555 goat anti-Rabbit (green fluorescence) secondary antibody (1:5000, Molecular Probes^®^, A21428) at room temperature for 1 h. After counterstaining with DAPI (CAT# 1306, Molecular Probes) for 30 min, slides were examined under a Zeiss LSM780-UV and LSM880-UV meta confocal microscope (Carl Zeiss Inc.) using a Plan-Apochromat 40′/1.3 Oil DIC objective. Fluorescence intensities and percent of positive cells were measured by ImageJ/Fiji (NIHV 2.9.0/1.54b, National Institutes of Health, USA).

### 4.9. Migration/Scratch Assay

We seeded HPV-16 Ect1/E6E7 cells in 12-well plates (three repeats per sample) at a concentration of 0.3 × 10^6^ cells/well and cells were incubated at 37 °C and 5% CO_2_ chambers until they reached 80% of confluence. Cell cultures were scratched with a 200 μL sterile pipette tip and then detached cells were washed away with PBS (1×). Next, 1 mL of DMSO 0.1% (vehicle control) or CSC (3R4F (10^−3^ or 10 μg/mL)) was added independently to plates. Horizontal reference lines were made on the bottom of each plate with an ultrafine tip marker to create a grid for alignment to obtain the same field for each image acquisition run. Zeiss AxioObserver epifluorescence microscope (Carl Zeiss Inc., Oberkochen, Germany) was used, with the reference marks as a guide. Selected regions of interest were imaged using an EC Plan-Neofluar 10×/0.3 Ph1 objective every 3 h for 72 h. We determined the scratch area, wound coverage of the total area, and mean and standard error of the scratch width with the aid of a plugin under Labkit in ImageJ/Fiji(NIHV 2.9.0/1.54b, National Institutes of Health, USA).We calculated the percentage of wound closure according to an area method [24,25].

### 4.10. RNA Extraction and RT^2^ Profiler Array

Total RNA was isolated by Trizol reagent (Invitrogen). A total of 1 μg of RNA was used for reverse transcription according to the manufacturer’s instructions (ABM, 8385 St George St, Vancouver, BC V5X 4P3, Canada). The quantitative real-time PCR (qRT-PCR) was performed using the Power SYBR Green Master Mix (Applied Biosystems, Foster City, CA, USA) and RT^2^ Profiler PCR Array kit (384-well [4 × 96] format) for human epithelial to mesenchymal transition (Qiagen; PAHS-090ZA, Hilden, Germany). RNA was extracted from HPV-16 Ect1/E6E7 cells exposed to their appropriate concentrations of CSC (3R4F (10^−3^, and 10 μg/mL)) or DMSO 0.1% (vehicle control) for 72 h using RNeasy Mini Kit (Qiagen, Germantown, MD, USA) and then purified followed by reverse-transcription to cDNA using a cDNA RT^2^ First Strand Kit (Qiagen, USA). Real-time PCR was run on ABI Quantstudio 7 Flex (ThermoFisher Scientific, Waltham, MA, USA). For each array, there were 84 related genes and three replicates of samples for each group. The data were analyzed by RT^2^ profiler PCR array analysis tool and Ingenuity Pathway Analysis (IPA, Qiagen, Hilden, Germany).

### 4.11. RTK Phospho-Protein Array

Protein extractions obtained from vehicle- and CSC-treated cells at 72 h were used to conduct RTK phospho-protein array analysis (R&D Systems, CAT# ARY001B, Minneapolis, MN, USA), with each array containing 49 specific antibodies for human RTKs. There were two replicates for each antibody. The array was conducted following the manufacturer’s instructions to detect phosphorylated tyrosine sites on RTKs (#ARY001B, R&D Systems, Minneapolis, MN, USA). Band intensity was quantified by ImageJ/Fiji (NIHV 2.9.0/1.54b, National Institutes of Health, USA). Data represents a composite of four replicates.

### 4.12. Pathway and Network Analysis by Ingenuity Pathway Analysis (IPA)

The list of differentially expressed genes in the RT^2^ profiler PCR arrays containing gene identifiers and corresponding expression values was uploaded into the IPA software (V111725566 IPA 2023 Q4 release) analysis program (Qiagen, Hiden, Germany). The “core analysis” was used to interpret the differentially expressed gene data, which included canonical pathways, upstream regulators, associated diseases, and functions, as well as molecule networks.

### 4.13. Statistical Analysis

The results were expressed as mean ± SE of at least three replicates. The statistical significance of the differences was determined by a *t*-test or one-way analysis of variance (ANOVA) followed by Dunnett’s post hoc comparisons test. Statistical significance was defined as *p* < 0.05 (*) or *p* < 0.01 (**). To analyze the results of the H&E staining, we used the reference of circularity measurements to quantify the elongated cells and the total number of cells [23]. Data were reported to Microsoft Excel to perform Paired *t*-tests to compare the difference between the elongated cells and the total number of cells in each group compared to the control. Additionally, to analyze the results of the scratch assay, we used a two-way ANOVA and Bonferroni tests to perform multiple comparisons. We performed paired *t*-tests to compare the difference between the area and the average length obtained with respect to the manual measurement and the other available macros in ImageJ/Fiji (NIHV 2.9.0/1.54b, National Institutes of Health, USA). [24].

## Data Availability

The data that support the findings of this study are available upon request.

## Supplementary Materials

The following supporting information can be downloaded at: https://www.mdpi.com/article/10.3390/ijms25094902/s1.
