# Supplementary material for: Tobacco Smoke Condensate Induces Morphologic Changes in Human Papillomavirus-Positive Cervical Epithelial Cells Consistent with Epithelial to Mesenchymal Transition (EMT) with Activation of Receptor Tyrosine Kinases and Regulation of TGFB
Source: Int J Mol Sci. 2024 Apr 30;25(9):4902. doi: 10.3390/ijms25094902 (PMC11084578; doi:10.3390/ijms25094902)
Supplement: Supplementary file 1 [file ijms-25-04902-s001.zip › ijms-2954198-supplementary.pdf]

Table S1. Differential expression of genes in CSC-exposed (10 μg/mL) versus vehicle control cells.

Symbol	Entrez Gene Name	Expr *p*-Value	Expr Fold Change	Location
FOXC2	forkhead box C2	3.26E-02	12.10	Nucleus
CDH2	cadherin 2	1.69E-05	6.34	Plasma Membrane
SNAI1	snail family transcriptional repressor 1	8.45E-03	2.80	Nucleus
CTNNB1	catenin beta 1	8.23E-05	2.66	Nucleus
SNAI2	snail family transcriptional repressor 2	1.65E-03	2.60	Nucleus
FN1	fibronectin 1	1.38E-04	2.39	Extracellular Space
TGFB2	transforming growth factor beta 2	1.54E-02	2.30	Extracellular Space
SMAD2	SMAD family member 2	1.54E-03	2.13	Nucleus
PDGFRB	platelet derived growth factor receptor beta	1.81E-04	2.08	Plasma Membrane

Note: This table was derived from IPA Canonical Pathway analysis on RT² EMT Profiler Array.
Red = Upregulated gene expression fold changes.
